# From nutrition knowledge to sustainable diets: a cross-sectional serial mediation model of dietary self-efficacy and mindful eating

**DOI:** 10.3389/fnut.2026.1812781

**Published:** 2026-05-15

**Authors:** Uğur Caba, Ayşe Sena Çakir, Mehmet Behzat Turan, Osman Pepe, Aydın Pekel, Alper Bahçe, Gül Bahar Bayiroğlu, İbrahim Dalbudak

**Affiliations:** 1 Faculty of Sports Sciences, İstanbul Gelişim University, İstanbul, Türkiye; 2 Department of Health Management, Develi Faculty of Social and Human Sciences, Kayseri University, Kayseri, Türkiye; 3 Department of Recreation, Faculty of Sports Sciences, Erciyes University, Kayseri, Türkiye; 4 Faculty of Sports Sciences, Süleyman Demirel University, Isparta, Türkiye; 5 Department of Sports Management, Faculty of Sports Sciences, Marmara University, Istanbul, Türkiye; 6 Department of Private Security and Protection, Bunyan Vocational School, Kayseri University, Kayseri, Türkiye; 7 Institute of Health Sciences, Erciyes University, Kayseri, Türkiye; 8 Department of Sports Management, Faculty of Sport Sciences, Süleyman Demirel University, Isparta, Türkiye; 9 Department of Sports Management, Faculty of Sports Sciences, Uşak University, Uşak, Türkiye

**Keywords:** nutrition knowledge, diet self-efficacy, mindful eating, sustainable nutrition, healthy eating behaviors

## Abstract

**Background:**

Sustainable and healthy eating plays a crucial role in reducing environmental impact and protecting long-term human health. However, the development of such dietary behaviors depends not only on general awareness but also on individuals’ capacity to translate nutritional knowledge into consistent, actionable food choices. Understanding how this transformation occurs is particularly important for young adults, who are in a formative stage of establishing lifelong eating habits. Examining the psychological mechanisms that facilitate the conversion of knowledge into behavior may help promote sustainable, healthy dietary practices in this age group.

**Objective:**

This study aimed to examine the serial mediating roles of diet self-efficacy and mindful eating in explaining how nutrition knowledge is translated into sustainable and healthy eating behaviors among sports science students. The research aimed to elucidate the widely discussed knowledge–behavior gap in dietary practices through its underlying psychological mechanisms.

**Methods:**

The study used a correlational research design and included 671 sports science students. Data were collected using the Nutrition Knowledge Scale, Diet Self-Efficacy Scale, Mindful Eating Scale, and the Sustainable and Healthy Eating Behaviors Scale. Hypotheses were tested using a serial mediation analysis (Model 6) with Hayes’ PROCESS Macro.

**Results:**

Nutrition knowledge was significantly associated with sustainable and healthy eating behaviors both directly and indirectly. While nutrition knowledge positively predicted diet self-efficacy and mindful eating, diet self-efficacy was negatively associated with mindful eating. Furthermore, the serial indirect effect through diet self-efficacy and mindful eating was negative, suggesting a more complex, non-linear mechanism than initially hypothesized. These findings suggest the presence of potentially competing self-regulatory processes rather than a purely facilitative pathway. The serial mediation pathway was statistically significant.

**Conclusion:**

The findings indicate that nutrition knowledge alone is insufficient; self-efficacy beliefs and mindfulness-based self-regulation processes are critical for transforming knowledge into behavior. These results suggest that interventions aimed at improving sustainable and healthy eating behaviors should integrate cognitive expertise with the development of psychological skills.

## 1 Introduction

The escalating global climate crisis, depletion of natural resources, and the growing burden of diet-related chronic diseases have made it imperative to reconsider food consumption patterns across both health and environmental dimensions (1, 2). The substantial impacts of food systems on greenhouse gas emissions, biodiversity loss, and water use necessitate linking individual dietary choices to sustainable development goals (3, 4). In this context, sustainable and healthy diets are conceptualized as a holistic approach encompassing dietary patterns that are low in environmental impact, accessible, safe, and culturally acceptable (2, 5). Within the United Nations’ 2030 Agenda, the promotion of responsible consumption and production patterns underscores the importance of disseminating sustainable dietary behaviors across societies (6).

The adoption of sustainable and healthy eating behaviors is strongly influenced by individual determinants (7, 8). Among these determinants, nutrition knowledge is regarded as a fundamental cognitive resource that enables individuals to make informed choices about food composition and relationships with the health environment (9, 10). However, previous research indicates that increases in nutrition knowledge do not consistently lead to direct behavioral change, revealing a systematic gap between knowledge and practice (7, 8). This discrepancy highlights the need for developing models that examine the role of psychosocial mechanisms in translating nutrition knowledge into sustainable dietary behaviors (11, 12).

Diet self-efficacy, a prominent concept in the behavior change literature, reflects individuals’ perceived capability to initiate and maintain healthy eating behaviors (12, 13). Individuals with high levels of self-efficacy have been reported to be more consistent in adopting and sustaining healthy dietary practices and to demonstrate more effective diet-related self-management behaviors (14, 15). Within the framework of Social Cognitive Theory, self-efficacy is proposed to function as a central psychological mediator in the relationship between knowledge and behavior (16). Accordingly, nutrition knowledge has been suggested to be associated with diet self-efficacy and, through this psychological construct, may be linked to dietary behaviors (7, 17).

In recent years, the mindful eating approach has emerged as an effective strategy for regulating eating behaviors (18, 19). Mindful eating has been reported to enhance individuals’ sensitivity to hunger–satiety cues and to reduce automatic consumption patterns (20). Studies among university students indicate that mindful eating is associated with higher diet quality and a more plant-based dietary pattern (21, 22). Furthermore, recent reviews emphasize that mindful eating should be considered a psychological mechanism that supports sustainable dietary behaviors (23, 24).

Within the scope of sustainability science, dietary behaviors represent a critical intersection between individual health, environmental integrity, and responsible resource use. Sustainable healthy eating directly contributes to several United Nations Sustainable Development Goals, particularly SDG 2 (Zero Hunger), SDG 3 (Good Health and Well-Being), SDG 12 (Responsible Consumption and Production), and SDG 13 (Climate Action). Understanding the psychological mechanisms that enable individuals to translate nutritional knowledge into sustainable dietary behaviors is, therefore, not only a public health concern but also a sustainability imperative. By focusing on sport science students who will become future educators, trainers, and decision-makers in health-related domains, this study positions dietary behavior change as a leverage point for long-term sustainability transitions.

Within this theoretical and empirical framework, sustainable and healthy eating behaviors cannot be explained by a single variable; instead, comprehensive models are needed that examine how nutrition knowledge is translated into behavior through sequential psychological processes, such as diet self-efficacy and mindful eating (25). Sports science students who have the potential to serve as role models in health and performance domains are considered a strategic sample for examining these relationships (26). Accordingly, the present study aims to investigate the association between nutrition knowledge and sustainable and healthy eating behaviors among sports science students within a serial mediation model in which diet self-efficacy and mindful eating operate sequentially.

Despite the growing body of literature examining the determinants of sustainable and healthy eating behaviors, the mechanisms through which nutrition knowledge is translated into behavior remain insufficiently understood. In particular, limited research has simultaneously examined sequential psychosocial processes that may explain this relationship within a unified analytical framework.

Accordingly, the present study contributes to the literature by testing a theoretically grounded serial mediation model integrating diet self-efficacy and mindful eating as complementary self-regulatory mechanisms. This approach provides a more nuanced explanation of the knowledge–behavior gap by demonstrating how cognitive resources can be translated into behavioral outcomes through interconnected psychological pathways (16, 25).

It is important to note that, given the cross-sectional and correlational design of the present study, the proposed model reflects statistical associations rather than causal relationships. Although the hypothesized pathways are theoretically grounded, causal inferences cannot be drawn, and the directionality of effects should be interpreted with caution (25, 27).

### 1.1 Nutrition knowledge

Nutrition knowledge refers to individuals’ cognitive awareness of dietary recommendations, food composition, everyday food choices, and relationships between diet and disease. According to Parmenter and Wardle, nutrition knowledge is a multidimensional construct that encompasses not only theoretical understanding but also the ability to apply this knowledge to daily food selections (9). Nutrition knowledge level also reflects individuals’ capacity to accurately comprehend and interpret fundamental principles of healthy eating, food groups, and dietary guidelines. However, the literature indicates that nutrition knowledge alone is insufficient for the acquisition and maintenance of healthy eating behaviors, highlighting the decisive role of psychosocial factors in translating knowledge into action (10, 28).

Within the framework of Bandura’s Social Cognitive Theory, knowledge is emphasized as an important cognitive resource that strengthens individuals’ self-efficacy perceptions, while enhanced self-efficacy plays a decisive role in the adoption and maintenance of healthy behaviors (29). Nutrition education-based studies indicate that increases in nutrition knowledge are associated with the development of self-efficacy in areas such as meal planning, healthy grocery shopping, food preparation, and food selection (30, 87). Moreover, higher levels of nutrition knowledge support individuals’ acquisition of new culinary skills, use of healthy cooking techniques, and increased fruit and vegetable consumption, with strengthened self-efficacy playing a pivotal role in this process (31). Alptekin and Duman (17) reported a positive and significant relationship between nutrition knowledge related to weight management and diet self-efficacy, indicating that as nutrition knowledge increases, individuals’ self-efficacy beliefs regarding their ability to sustain diet-related behaviors also rise.

### 1.2 Diet self-efficacy

Self-efficacy is defined as an individual’s belief in their capability, competence, and confidence to perform a given behavior (29). It is also considered a strong determinant of health behaviors (13). Diet self-efficacy refers to individuals’ confidence in their ability to utilize the skills required to engage in healthy eating behaviors (15). This confidence is shaped not only by knowledge but also by psychological, social, and behavioral experiences (32).

While diet self-efficacy reflects individuals’ confidence in maintaining healthy dietary practices, mindful eating plays an important role in transforming this confidence into conscious food choices. Mindfulness-based dietary interventions have been reported to improve both diet self-efficacy and diet quality (33). In a randomized controlled trial conducted by Miller et al. (34) among individuals with type 2 diabetes, a mindful eating–based intervention was shown to increase cognitive control over eating behaviors, reduce tendencies toward uncontrolled eating, and strengthen diet-related self-efficacy perceptions. The study further indicated that improvements in mindful eating contributed to more accurate recognition of hunger and satiety cues and to the deliberate regulation of eating behaviors, which in turn supported the development of diet self-efficacy. These findings suggest that mindful eating is not merely an awareness skill but also a key psychosocial mechanism that promotes the sustainability of healthy eating behaviors (34).

### 1.3 Mindful eating

Mindful eating refers to an eating approach that focuses not on what is eaten but on how and why eating behavior occurs, internalizing physical hunger satiety cues, becoming aware of the influence of emotions and thoughts, avoiding environmental distractions, and attending nonjudgmentally to the food being consumed in the present moment. The core of mindful eating involves full awareness of the eating experience, including the taste and texture of food (35, 36). It encompasses developing awareness of physical and psychological hunger and fullness signals and making healthier food choices in response to these cues. Mindful eating has also been conceptualized as being present while eating, paying attention to the sensory aspects of food, and distinguishing between physical and emotional sensations that trigger eating (37).

Research indicates that mindful eating supports healthy and conscious eating behaviors (19, 38). In this context, a study among children found that the mindful eating awareness subdimension was positively associated with adherence to the Mediterranean diet (39). Another study conducted with adults reported that the eating discipline dimension of mindful eating particularly supported adherence to the Mediterranean diet and healthy dietary behaviors (21). In a large-scale study among young adults in Türkiye, adherence to the Mediterranean diet was positively associated with intuitive and mindful eating; individuals with higher adherence exhibited significantly higher mindful eating scores, and this relationship remained robust after controlling for body mass index and lifestyle variables. These findings suggest that mindful eating may be associated with dietary patterns considered relatively sustainable from an environmental perspective (40). Similarly, a study among university students demonstrated that mindful eating dimensions, such as sensitivity to planetary health and to hunger satiety cues, were associated with healthy plant-based dietary patterns (22). Indeed, the review by Pompili and Carfora (23) synthesized these findings within a comprehensive framework, emphasizing that mindful eating should be regarded not only as a behavior regulation strategy related to individual health but also as a potential psychological mechanism that can support sustainable dietary behaviors.

Despite growing interest in sustainable behaviors in the literature, the role of mindfulness in environmentally impactful individual behaviors, such as food choices, has been examined to a limited extent (41). Most existing studies rely on cross-sectional designs (42, 43) and have focused on the associations between mindfulness and both intentional pro-environmental behaviors and dietary patterns with positive environmental outcomes, such as the Mediterranean diet. However, empirical evidence on mindfulness-based interventions remains limited, and the effects of these approaches on promoting sustainable food consumption appear to vary across contexts (44).

### 1.4 Sustainable and healthy eating behaviors

Although various initiatives have been undertaken to define the core principles of sustainable and healthy diets, there is still no consensus on the scope of these concepts (5, 45, 46). According to the Food and Agriculture Organization and the World Health Organization (WHO), sustainable healthy diets are defined as “dietary patterns that promote all dimensions of individuals’ health and well-being; have low environmental pressure and impact; are accessible, affordable, safe, and equitable; and are culturally acceptable” (2). Sustainable diets are characterized by low environmental impact and by being accessible, cost-effective, safe, equitable, and culturally acceptable. The guiding principles for sustainable healthy diets proposed by the FAO and WHO encompass health-related aspects (minimally processed and balanced diets; whole grains, legumes, a variety of fruits and vegetables; reduced consumption of animal-based products; adequate energy and nutrient intake for growth and development; and reduced risk of diet-related noncommunicable diseases), environmental aspects (lower greenhouse gas emissions; reduced water and land use; pollution mitigation; biodiversity conservation; and minimizing the use of plastic-based packaging), and sociocultural dimensions (accessibility, cultural acceptance and culinary practices, and the reduction of food loss and waste) (47).

It is emphasized that the development of sustainable and healthy eating behaviors is shaped not only by environmental awareness but also by individual cognitive and psychosocial processes (48, 49). Nutrition knowledge, in particular, is regarded as a fundamental factor enhancing individuals’ capacity to evaluate nutritionally adequate food choices with lower environmental impact (9, 10). Nevertheless, the literature indicates that knowledge alone is insufficient to bring about behavioral change; instead, its influence is exerted through psychological mechanisms such as perceived control, self-efficacy, and mindfulness (16, 50).

Accordingly, Żakowska-Biemans et al. (46) demonstrated that sustainable and healthy eating behaviors consist of multidimensional patterns, including healthy food choices, reduced meat consumption, preferences for local and seasonal foods, and avoidance of food waste. Similarly, Springmann et al. (51) emphasized that restructuring healthy dietary patterns in alignment with environmental sustainability goals is critical for both public health gains and reductions in carbon footprints. Studies conducted among university students further indicate that environmental concern and healthy eating intentions often co-occur and jointly shape sustainable dietary behaviors (52).

Recent research has shown that self-efficacy plays an important role in maintaining healthy eating behaviors among young adults (88). As individuals’ confidence in their ability to make and sustain healthy food choices increases, they are more likely to adopt plant-based dietary patterns and environmentally low-impact diets. This relationship is supported by studies among university students demonstrating a positive association between self-efficacy and intentions to adopt plant-based diets (53). In addition, the mindful eating approach has been reported to be associated with healthier, more plant-based dietary patterns and, in this respect, to offer a psychological mechanism that may facilitate individuals’ shift toward environmentally lower-impact, sustainable eating practices (54).

Within this framework, the present study aims to examine the sequential mediating roles of diet self-efficacy and mindful eating in the association between sustainable and healthy nutrition knowledge and eating behaviors among sports science students.

### 1.5 The present study

This study is grounded in an integrative theoretical framework that combines Social Cognitive Theory, the Theory of Planned Behavior, and mindfulness-based approaches to explain how nutrition knowledge is translated into sustainable and healthy eating behaviors among sports science students. Although nutrition knowledge constitutes a critical cognitive foundation for healthy food choices, accumulating evidence indicates that knowledge alone is often insufficient to produce lasting behavioral change, thereby giving rise to the frequently discussed knowledge–behavior gap in health-related behaviors (7, 11, 12).

From a social cognitive perspective, self-efficacy is a central mechanism for translating knowledge into action. Diet self-efficacy reflects individuals’ perceived capability to initiate and maintain healthy eating behaviors in challenging situations and has been identified as a strong predictor of dietary adherence and self-regulation (12, 14). Individuals with higher levels of nutrition knowledge are expected to feel more competent in making conscious food choices and, consequently, to exhibit stronger perceptions of diet self-efficacy.

In parallel, mindful eating, grounded in mindfulness theory, emphasizes present-moment awareness, nonjudgmental attention to internal hunger satiety cues, and the reduction of automatic or emotional eating behaviors (19). A growing body of research has demonstrated that mindful eating is associated with healthier dietary patterns, enhanced self-regulation, and sustainable eating practices (55, 56). In this context, self-efficacy is theoretically proposed to support mindful eating by enabling individuals to exert stronger cognitive and emotional control over eating-related decisions.

Within this integrative perspective, nutrition knowledge is assumed to influence sustainable and healthy eating behaviors not only directly but also indirectly through sequential psychological mechanisms involving diet self-efficacy and mindful eating.

Based on the proposed theoretical framework, this study conceptualizes a serial mediation model in which the association between nutrition knowledge and sustainable and healthy eating behaviors is transmitted through two sequential mediators: diet self-efficacy (M1) and mindful eating (M2). Accordingly, higher levels of nutrition knowledge are expected to strengthen diet self-efficacy by increasing individuals’ confidence in their ability to maintain healthy eating behaviors. Enhanced diet self-efficacy, in turn, is assumed to promote more conscious, self-regulated, and mindfulness-based eating practices, thereby increasing mindful eating. In the final stage, mindful eating is hypothesized to reduce impulsive consumption, support environmentally and nutritionally responsible food choices, and ultimately contribute to sustainable, healthy eating behaviors.

This sequential process is consistent with contemporary mediation theories, which emphasize that behavioral outcomes often emerge through interconnected psychological mechanisms rather than single mediators (25). Accordingly, diet self-efficacy and mindful eating are posited to function as serial mediators in explaining the translation of nutrition knowledge into sustainable, healthy eating behaviors.

This study offers several theoretical contributions to the literature on eating behavior and sustainability. First, by empirically addressing the knowledge–behavior gap through a serial mediation model, it demonstrates that nutrition knowledge alone is insufficient to explain sustainable, healthy eating behaviors. Second, by integrating diet self-efficacy and mindful eating within a unified explanatory framework, the study bridges social cognitive and mindfulness-based approaches and provides a more comprehensive perspective on mechanisms of dietary behavior change. Third, it extends existing literature by positioning mindful eating not merely as an outcome of awareness but as a critical sub-mechanism activated by self-efficacy beliefs. Finally, by focusing on sports science students who are expected to possess relatively high levels of health literacy, the study provides novel insights into the role of psychological processes in shaping behavior even among individuals with substantial knowledge. Collectively, these contributions underscore the need for multistage psychological pathways to explain sustainable and healthy eating behaviors, thereby advancing theory development and providing a conceptual foundation for future intervention research.

This study contributes to the field by providing a theoretically grounded explanation of the knowledge behavior gap in sustainable nutrition through the integration of diet self-efficacy and mindful eating within a serial mediation framework. Rather than assuming a direct link between knowledge of behavior and its enactment, the findings highlight the importance of self-regulatory mechanisms in translating cognitive resources into practice.

However, the results should be interpreted within the limitations of self-reported data and cross-sectional design, and therefore do not imply causal relationships.

Based on the integrative theoretical framework combining Social Cognitive Theory and mindfulness-based approaches, the present study proposes a set of hypotheses examining both direct and indirect associations among the study variables. Given the mixed empirical evidence regarding the interplay between control-based and awareness-based self-regulatory processes, certain pathways are conceptualized as non-directional and exploratory. In addition, gender is included as a covariate to control for potential differences in dietary behaviors and related psychological processes.

*H1*: Nutrition knowledge is positively associated with sustainable and healthy eating behaviors.

*H2*: Nutrition knowledge is positively associated with diet self-efficacy.

*H3*: Nutrition knowledge is positively associated with mindful eating.

*H4*: Diet self-efficacy is positively associated with sustainable and healthy eating behaviors.

*H5*: Mindful eating is positively associated with sustainable and healthy eating behaviors.

*H6*: Diet self-efficacy is associated with mindful eating; however, the direction of this relationship is treated as exploratory due to potential divergence between control-based and awareness-based self-regulation processes.

*H7*: Diet self-efficacy mediates the association between nutrition knowledge and sustainable and healthy eating behaviors.

H8: Mindful eating mediates the association between nutrition knowledge and sustainable and healthy eating behaviors.

*H9*: Diet self-efficacy and mindful eating jointly operate as serial mediators in the association between nutrition knowledge and sustainable and healthy eating behaviors. Given the potential for suppression and competing mechanisms, the direction of the serial indirect effect is interpreted exploratorily.

*H10*: Gender is associated with sustainable and healthy eating behaviors and related psychological variables (diet self-efficacy and mindful eating), and its inclusion as a covariate may influence the strength and significance of the associations among the primary study variables.

This hypothesis structure reflects a contemporary approach to behavioral modeling, acknowledging that psychological mechanisms underlying eating behavior may not operate in a strictly linear or unidirectional manner. Instead, multiple self-regulatory processes may interact in complex and at times competing ways, particularly in cross-sectional designs (25, 57).

## 2 Materials and methods

### 2.1 Research model

This study employed a relational screening model to examine the relationship between variables. Relational screening aims to identify the extent to which two or more variables change in relation to each other and assess whether a significant connection exists based on this change. This non-experimental approach provides insights into the direction and strength of the relationship between variables, enabling potential predictions (58, 59).

### 2.2 Determination of sample size using Monte Carlo simulation

The adequacy of the sample size was determined using Monte Carlo simulation, which is considered one of the most robust methods for power estimation in complex models such as serial multiple mediation, where traditional analytical power formulas are limited (60, 61). This approach estimates statistical power by repeatedly generating data from predefined population parameters and evaluating the proportion of simulated samples that yield statistically significant effects.

In the present study, the Monte Carlo simulation was specified according to the hypothesized serial mediation model (PROCESS Model 6), in which nutrition knowledge (X) is associated with sustainable and healthy eating behaviors (Y) through dieting self-efficacy (M1) and mindful eating (M2). Population parameters for the simulation were informed by effect sizes reported in previous mediation research in nutrition, health psychology, and behavioral sciences, and conservative path coefficients were adopted to avoid inflation of statistical power (25, 62).

The simulation procedure involved generating 10,000 replications for varying sample sizes. Indirect effects were evaluated using bias-corrected bootstrap confidence intervals with 5,000 resamples, and statistical power was defined as the proportion of simulated samples in which the 95% confidence interval for the indirect effects did not include zero (63).

The Monte Carlo results indicated that a minimum sample size of approximately *N* = 220 was required to achieve an acceptable statistical power level of 0.80 for detecting the hypothesized serial indirect effects. Sample sizes above this threshold yielded progressively higher power estimates, with diminishing marginal gains beyond *N* ≈ 400.

Given that the final study sample consisted of 671 participants, the achieved sample size substantially exceeded the minimum required sample size determined by the Monte Carlo simulation. This ensured high statistical power (>0.95) for detecting both direct and indirect effects, enhanced the precision of parameter estimates, and increased the robustness and generalizability of the findings. Therefore, the sample size was deemed more than sufficient for testing the proposed serial mediation model.

### 2.3 Participants

This study was conducted among students enrolled in the Faculties of Sports Sciences at universities in Türkiye. The sample consisted of 671 sports science students studying during the 2024–2025 academic year in the departments of physical education and sports teaching, coaching education, sports management, and recreation at different universities. Participants were recruited using convenience sampling.

Participants were recruited through classroom announcements and the distribution of an online survey across multiple universities. The survey was administered via Google Forms, allowing participants to complete it at their convenience. Students were given sufficient time to review and respond to all items without time pressure, ensuring more thoughtful and accurate answers. Participation was entirely voluntary, and no incentives were offered for taking part in the study.

Prior to data collection, students were informed about the purpose and scope of the research as well as confidentiality principles, and written informed consent was obtained. The study was conducted in accordance with the principles of the Declaration of Helsinki.

#### 2.3.1 Inclusion criteria

The criteria for inclusion in the study were as follows:

- Being an actively enrolled undergraduate student in one of the departments within the Faculty of Sports Sciences (physical education and sports teaching, coaching education, sports management, or recreation),
- Being 18 years of age or older,
- Possessing sufficient Turkish literacy to understand and respond to the measurement instruments used in the study,
- Agreeing to participate voluntarily and providing informed consent,
- Completing the survey forms fully and consistently.

#### 2.3.2 Exclusion criteria

Individuals with the following characteristics were excluded from the study:

- Being enrolled in a faculty or department other than the Faculty of Sports Sciences,
- Being under 18 years of age,
- Completing the questionnaire in an incomplete, random, or inconsistent manner,
- Declining to participate or failing to provide informed consent,
- Participants were identified as having submitted responses more than once during data collection.

### 2.4 Study model

This study aims to examine the relationship between nutrition knowledge level and sustainable and healthy eating behaviors among sport sciences students and to reveal the serial mediating roles of dieting self-efficacy and mindful eating in this relationship.

In serial multiple mediation, the association of an independent variable on a dependent variable is transmitted through multiple mediators in a specific causal sequence. This model allows researchers to test whether mediators function as part of a causal chain linking the predictor to the outcome (25).

Serial mediation models allow the examination of mechanisms operating in a theoretically specified sequence, in which one mediator influences another in a causal chain linking the independent and dependent variables (25). In the present study, diet self-efficacy is conceptualized as an initial self-regulatory mechanism that enhances individuals’ perceived control, which, in turn, facilitates mindful eating as a higher-order, awareness-based process.

**Figure 1:**
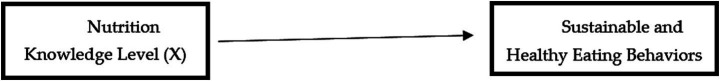
The association between nutrition knowledge level and sustainable and healthy eating behaviors.

**Figure 2:**
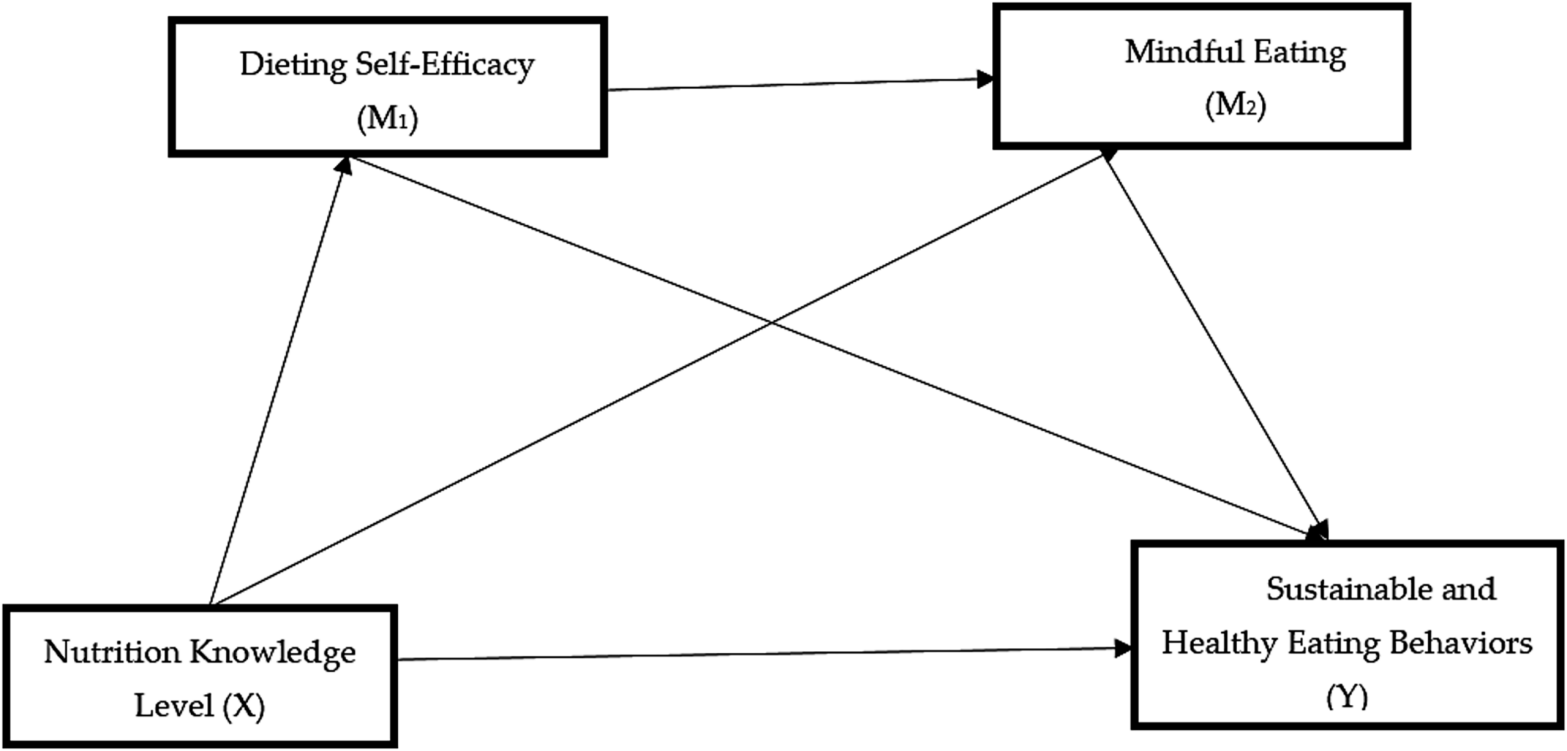
The serial mediation association between dieting self-efficacy and mindful eating.

Accordingly, the serial mediation effects of dieting self-efficacy (M1) and mindful eating (M2) in the relationship between nutrition knowledge level (X) and sustainable and healthy eating behaviors (Y) were examined in the data analysis. Figure 1 shows the association between nutrition knowledge level and sustainable and healthy eating behaviors, and Figure 2 shows the serial mediation effects of dieting self-efficacy and mindful eating.

### 2.5 Data collection tools

#### 2.5.1 Personal information form

A personal information form comprising six variables was prepared to collect demographic and professional data from participants. These variables include gender, age, field of study, income level, current residence, and weekly study hours.

In Table 1, 295 participants (44.0%) are female, and 376 participants (56.0%) are male. Regarding age groups, 180 participants (26.8%) are between 18 and 19 years old, 122 (18.2%) are between 20 and 21 years old, 112 (16.7%) are between 22 and 23 years old, and 188 (28.0%) are 24 years old and above. Regarding the field of study, 171 participants (25.5%) are in physical education and sport, 175 (26.1%) are in coaching education, 169 (25.2%) are in sport management, and 156 (23.2%) are in recreation. Concerning income level, 180 participants (26.8%) earned 0–2,999 TL, 122 participants (18.2%) earned 3,000–5,999 TL, 112 participants (16.7%) earned 6,000–8,999 TL, 104 participants (15.5%) earned 9,000–11,999 TL, and 153 participants (22.8%) earned 12,000 TL and above. Regarding place of residence, 207 participants (30.8%) lived in a dormitory, 216 participants (32.2%) in an apartment, and 248 participants (37.0%) with their family. Regarding the weekly working hours, 304 participants (45.3%) worked 1–9 h, 202 participants (30.1%) worked 10–19 h, and 165 participants (24.6%) worked 20 h or more.

**Table 1:** Descriptive statistics: frequency and percentage values.

Variable	Group	*N*	*f*	%
Gender	Female	671	295	44.0
	Male		376	56.0
Age	18–19	671	180	26.8
	20–21		122	18.2
	22–23		112	16.7
	24 ve üzeri		188	28.0
Field of study	Physical Education and Sport	671	171	25.5
	Coaching Education		175	26.1
	Sport Management		169	25.2
	Recreation		156	23.2
Income level	0–2,999	671	180	26.8
	3,000–5,999		122	18.2
	6,000–8,999		112	16.7
	9,000–11,999		104	15.5
	12,000 and above		153	22.8
Current residence	Dormitory	671	207	30.8
	Apart		216	32.2
	Family		248	37.0
Weekly study hours	1–9	671	304	45.3
	10–19		202	30.1
	20 and above		165	24.6

#### 2.5.2 The Nutrition Knowledge Level Scale for adults (NKLSA)

In this study, the Nutrition Knowledge Level for Adults Scale (YETBİD) was used to assess participants’ nutrition knowledge levels. The scale was developed by Batmaz (64), and its validity and reliability were established. It consists of 20 items under the domain of Basic Nutrition and Food Health Knowledge and is rated on a 5-point Likert scale.

Items are scored as follows: “Strongly agree” (4 points), “Agree” (3 points), “Neither agree nor disagree” (2 points), “Disagree” (1 point), and “Strongly disagree” (0 points). Items 1, 3, 6, 8, 13, 16, 19, and 20 are reverse-coded.

The maximum possible score on the scale is 80, with higher total scores indicating greater nutrition knowledge and lower scores indicating poorer nutrition knowledge. Based on total scores, participants’ nutrition knowledge levels are classified as “poor” (below 45 points), “moderate” (45–55 points), “good” (56–65 points), and “very good” (65 points and above).

In the scale development study, the internal consistency coefficient (Cronbach’s alpha) was reported as 0.74.

#### 2.5.3 The Dieting Self-Efficiency Scale (DIET-SE)

In this study, the Diet Self-Efficacy Scale (DSES) was employed to assess individuals’ perceived self-efficacy in maintaining dietary behaviors. The original version of the scale, titled the Dieting Self-Efficacy Scale (DIET-SE), was developed by Stich et al. (65), and its Turkish adaptation was conducted by Hamurcu et al. (66). The scale measures individuals’ beliefs about their ability to adhere to their diets under various environmental and emotional conditions.

The scale consists of 11 items and is scored on a 5-point Likert scale (1 = Not at all confident, 5 = Very confident). It comprises three sub-dimensions: High-calorie tempting foods (4 items), social and internal factors (4 items), and adverse emotional events (3 items). Total scores range from 11 to 55, with higher scores indicating greater levels of diet-related self-efficacy.

Internal consistency analyses revealed a total Cronbach’s alpha coefficient of 0.90, while the coefficients for the sub-dimensions were reported as 0.769, 0.812, and 0.791, respectively. These findings indicate that the Turkish version of the Diet Self-Efficacy Scale is a valid and reliable measurement instrument.

#### 2.5.4 The mindful eating questionnaire (MEQ)

To assess individuals’ levels of awareness regarding eating behaviors, the Turkish version of the Mindful Eating Questionnaire (MEQ-30), initially developed by Framson et al. (18) and adapted into Turkish by Köse et al. (36), was used. The scale was re-evaluated for cultural and linguistic appropriateness through expert consultation; items with potential for misunderstanding were removed, and new items were added, resulting in a revised 30-item structure.

The scale is rated on a 5-point Likert format (1 = never, 2 = rarely, 3 = sometimes, 4 = often, 5 = always). The minimum possible score is 30, and the maximum is 150, with higher scores indicating greater levels of mindful eating.

Unlike the original version, the Turkish form consists of seven sub-dimensions:

- Disinhibition (loss of quantity and time control related to restraint),
- Emotional eating (eating in response to emotional hunger, to feel good, or for satisfaction),
- Eating control (ability to regulate eating speed and maintain eating behavior),
- Eating discipline (planning, preparation, balancing, order, and time management),
- Focusing (attending to the taste of food during eating and disengaging from other thoughts or activities),
- Mindfulness (distinguishing physical hunger satiety cues, knowledge of food content, and awareness of healthy eating), and Interference (coping with sensory stimuli such as smell, sight, and sound, as well as distractions such as invitations, food variety, and advertisements).

The adaptation study was conducted with 318 volunteer university students, and the Cronbach’s alpha coefficient for the overall scale was reported as 0.733, indicating acceptable reliability.

#### 2.5.5 The Sustainable and Healthy Eating Behaviors Scale (SHEBS)

In this study, the SHEBS, initially developed by Żakowska-Biemans et al. (46) and adapted into Turkish by Köksal et al. (67), was used to assess participants’ sustainable and healthy eating behaviors.

The scale consists of seven factors and 32 items. These factors encompass quality labels, seasonal food consumption and avoidance of food waste, animal welfare, reduced meat consumption, healthy and balanced eating, local food consumption, and preference for low-fat foods.

Items are rated on a seven-point Likert-type scale, with responses ranging from never to always. The total scale score is calculated by averaging all factor scores. The possible range of total scores is 32 to 224, and increases in total and subscale scores indicate higher levels of sustainable and healthy eating behaviors. In the Turkish adaptation study, Cronbach’s alpha coefficients for the overall scale and its sub-dimensions ranged from 0.764 to 0.912, indicating good to excellent reliability. The Cronbach alpha values obtained from participants’ responses are presented in Table 2.

**Table 2:** Descriptive values of the subdimensions of the scales.

Scales	Number of items	Cronbach’s alpha
The Nutrition Knowledge Level	20	0.77
The Dieting Self-Efficacy	11	0.86
The Mindful Eating	30	0.84
The Sustainable and Healthy Eating Behaviors	32	0.94

When Table 2 is examined, Cronbach’s Alpha values indicate that the internal consistency coefficient for the nutrition knowledge level scale is 0.77, for the dieting self-efficacy scale is 0.86, for the mindful eating scale is 0.84, and the sustainable and healthy eating behaviors determination scale is 0.94.

Cronbach’s Alpha is a reliability coefficient used to assess the internal consistency of multi-item scales. In other words, it is used to determine the extent to which the items in a scale are related to each other, that is, whether they measure the same concept. The value range is 0 to 1; a value of 0.70 or higher is generally considered an acceptable level of internal consistency (68). These values demonstrate that the participants’ data on these scales exhibit an acceptable level of internal consistency.

### 2.6 Analysis of data

Data was analyzed using SPSS v22. The Kolmogorov–Smirnov test, one of the tests used to assess the normality of data distributions (69), was employed. The normality results of the scores obtained in this study are presented in Table 3.

**Table 3:** Skewness, Kurtosis, and Kolmogorov–Smirnov Test significance level results of the participants’ scale scores.

Scales	Skewness	Kurtosis
The Nutrition Knowledge	0.58	0.68
The Diet Self-Efficacy	0.11	−0.25
The Mindful Eating	0.53	0.94
The Sustainable Healthy Eating Behaviors	0.03	0.37

When Table 3 is examined, the skewness and kurtosis values of the data fall within ±1. Values within ±2 (70) are interpreted as indicating the absence of excessive deviations from normality. Consequently, the data were normally distributed and suitable for parametric tests. Pearson correlation analysis was used to determine the relationships between the variables. Regression analysis was used to determine the association between nutrition knowledge and sustainable healthy eating behaviors. To assess the serial mediation association between diet self-efficacy and mindful eating, regression analysis based on the indirect effect approach using the Bootstrap method was conducted via the PROCESS v3.5 macro. The PROCESS Macro Model 6 option developed by Hayes (25) was employed to examine the serial mediation effect, with a 5,000-resample option applied in the Bootstrap method. The 95% confidence interval values obtained from this analysis should not include zero (25, 71).

Given the cross-sectional nature of the data, the mediation model tested in this study reflects statistical associations rather than causal pathways. Therefore, the directionality of the relationships should be interpreted cautiously and within the bounds of theoretical plausibility (27).

All statistical associations are reported using non-causal terminology (e.g., “association,” “relationship,” “predictive association”) to reflect the correlational nature of the data.

## 3 Results

As shown in Table 4, the participating sports science students had a mean nutrition knowledge score of 63.92 ± 10.19, a mean diet self-efficacy score of 35.92 ± 9.39, a mean mindful eating score of 92.35 ± 14.20, and a mean sustainable and healthy eating behavior score of 132.88 ± 33.49.

**Table 4:** Descriptive statistics and Pearson correlation coefficients for the relationships between variables.

The Scales	Min.	Max.	M ± SD	1	2	3	4
Nutrition Knowledge^1^	39.00	99.00	63.92 ± 10.19	1			
Diet Self-Efficacy^2^	11.00	55.00	35.92 ± 9.39	0.78**	1		
Mindful Eating^3^	56.00	143.00	92.35 ± 14.20	0.71**	0.24**	1	
Sustainable and Healthy Eating Behaviors^4^	32.00	224.00	132.88 ± 33.49	0.49**	0.46**	0.39**	1

**p < 0.001, *n* = 671. 1, nutrition knowledge; 2, diet self-efficacy; 3, mindful eating; 4, sustainable and healthy eating behaviors.

In addition, significant positive correlations were observed among the study variables. Nutrition knowledge was strongly and positively correlated with diet self-efficacy (*r* = 0.78) and mindful eating (*r* = 0.71), and moderately and positively correlated with sustainable and healthy eating behaviors (*r* = 0.49). Diet self-efficacy showed a low but positive correlation with mindful eating (*r* = 0.24) and a moderate positive correlation with sustainable and healthy eating behaviors (*r* = 0.46). Mindful eating was also moderately and positively correlated with sustainable and healthy eating behaviors (*r* = 0.39).

As indicated in Table 5, the regression model indicates a significant relationship between nutrition knowledge and sustainable and healthy eating behaviors among sports science students [*F*(1,659) = 214.00, *p* < 0.001]. According to the *t*-test results for the significance of the regression coefficient, nutrition knowledge significantly predicted sustainable and healthy eating behaviors (*t* = 14.63, *p* < 0.001). This model accounted for 24% of the variance in sustainable and healthy eating behaviors (R^2^ = 0.24, *p* < 0.001).

**Table 5:** The association between nutrition knowledge and sustainable and healthy eating behaviors.

Variables		*β*	*SE*	*t*	*p*	*R*	*R* ^2^	*F*	*p*
Independent	Depend								
Nutrition Knowledge	SHB	1.62	0.11	14.63	0.00	0.49	0.24	214.00	0.00**

**p < 0.001.

**Figure 3:**
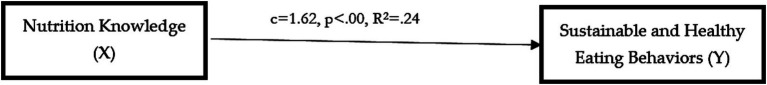
The association between nutrition knowledge and sustainable and healthy eating behaviors.

**Figure 4:**
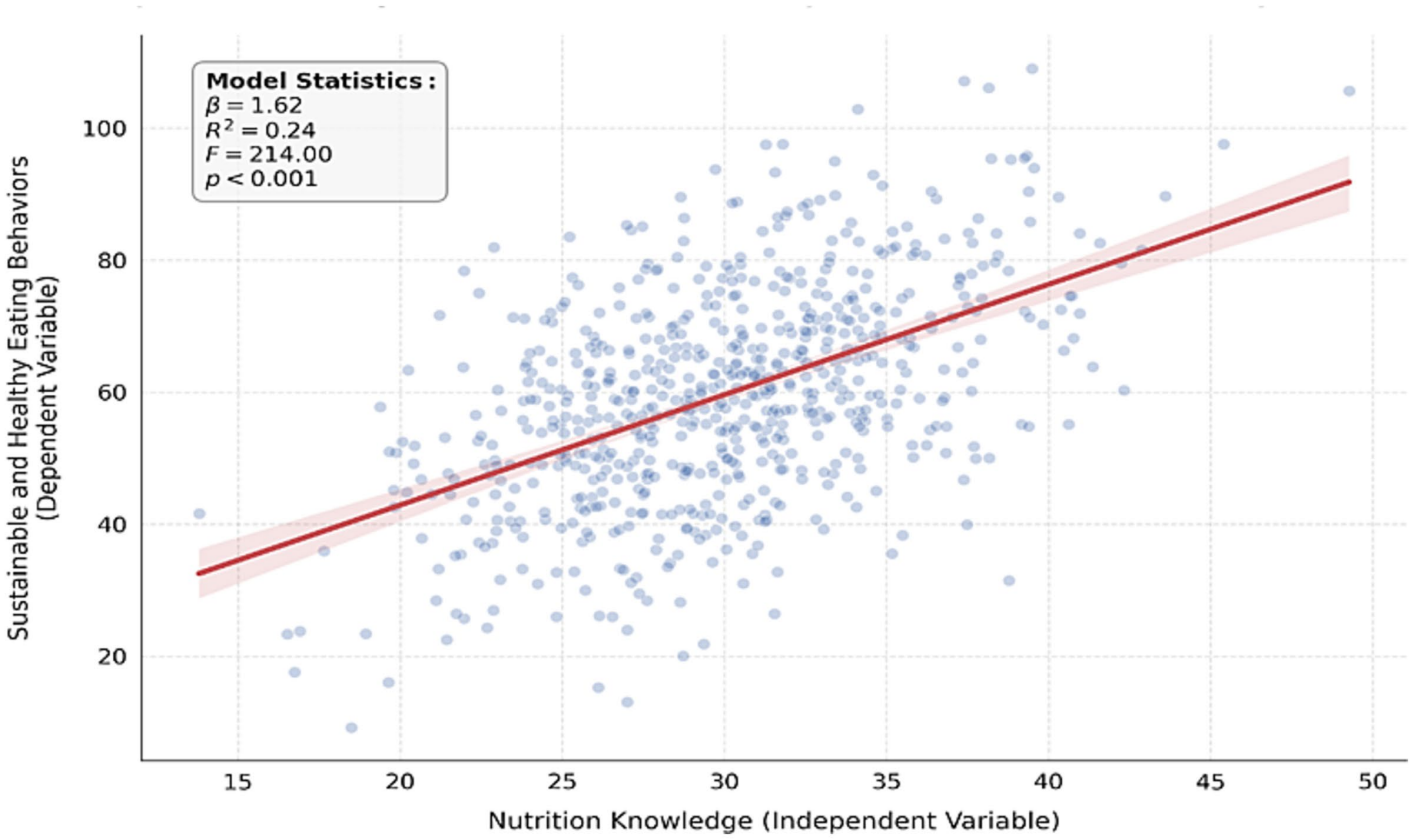
The dependent variable, sustainable and healthy eating behaviors, is a histogram.

Figure 3 illustrates the association between nutrition knowledge and sustainable and healthy eating behaviors among sports science students. In contrast, Figure 4 presents the histogram of the dependent variable, sustainable and healthy eating behaviors.

As shown in Table 6, the multicollinearity statistics for the regression model indicate that tolerance values were above 0.10 (ranging from 0.164 to 0.249). The variance inflation factor (VIF) values remained within acceptable limits (ranging from 4.020 to 5.634). These findings suggest that there were no powerful linear relationships among the independent variables and that the model did not suffer from serious multicollinearity. Accordingly, the regression coefficients can be interpreted as statistically reliable. Although VIF values remained within commonly accepted thresholds (<10), several values approached moderate levels (VIF > 5), suggesting potential multicollinearity. Given the strong correlations among predictors (*r* > 0.70), the possibility of suppression effects should be considered when interpreting regression coefficients (72).

**Table 6:** Collinearity statistics of predictor variables.

Model	Collinearity Statistics	
1	Tolerance	VIF
Diet Self-Awareness	0.197	5.066
Mindful Eating	0.249	4.020
Sustainable and Healthy Eating Behavior	0.164	5.634

As shown in Table 7, Cook’s Distance values were used to assess outliers and influential observations in the regression analysis. The results indicated that Cook’s Distance values ranged from 0 to 0.077, with a mean of 0.002 and a standard deviation of 0.005. These values were well below the commonly recommended critical threshold (Cook’s Distance < 1.00), suggesting that no observations were overly influential and that the regression results were not biased by any single observation. This supports the statistical reliability and stability of the obtained regression findings.

**Table 7:** Cook’s distance statistics.

Statistic	Min.	Max.	Mean	SD	*N*
Cook’s distance	0.000	0.077	0.002	0.005	671

As shown in Table 8, the regression analysis indicates a moderate relationship between the model and the dependent variable (*R* = 0.552). The coefficient of determination (R^2^ = 0.305) suggests that approximately 30.5% of the total variance in the dependent variable is explained by the independent variables included in the model. The adjusted R^2^ value (Adj. R^2^ = 0.301), which is very close to R^2^, indicates that the model is not biased by sample size and is structurally stable. In addition, the overall model was found to be statistically significant (*F* = 97.341, *p* < 0.001), demonstrating that the proposed regression model provides a good fit to the dataset.

**Table 8:** Regression model summary.

Model	*R*	*R* ^2^	Adjusted R^2^	Std. Error	*F*	*p*
1	0.552	0.305	0.301	27.99594	97.341	0.000**

**p < 0.01.

As shown in Table 9, the ANOVA results of the regression analysis indicate that the proposed model is statistically significant [*F*(3,667) = 97.341, *p* < 0.001]. The regression sum of squares (SS = 228,880.79) accounted for a substantial portion of the total variance (SS = 751,657.22), and compared with the residual sum of squares (SS = 522,776.43), the model demonstrated strong explanatory power. These findings indicate that the three independent variables in the model jointly explained a significant proportion of the variance in the dependent variable and that the regression model provided a good fit to the dataset.

**Table 9:** Analysis of Variance (ANOVA) for the regression model.

Model		Sum of squares	df	Mean square	*F*	*p*
1	Regression	228880.790	3	76293.597	97.341	0.000**
	Residual	522776.432	667	783.773		
	Total	751657.222	670			

**p < 0.01.

As shown in Table 10, the results of the multiple linear regression analysis indicate that the significant predictors of sustainable and healthy eating behavior were diet self-efficacy, mindful eating, and adult nutrition knowledge. Diet self-efficacy was found to have a positive and substantial effect on sustainable and healthy eating behavior (*B* = 1.938; *β* = 0.544; *t* = 7.479; *p* < 0.001). Similarly, mindful eating significantly and positively predicted sustainable and healthy eating behavior (*B* = 1.013; *β* = 0.429; *t* = 6.631; *p* < 0.001).

**Table 10:** Regression coefficients for predictors of sustainable and healthy eating behavior.

Model	Unstandardized coefficients	Standardized coefficients	*β*	*t*	*p*
1	*B*	Std. Error			
Diet Self-Awareness	1.938	0.259	0.544	7.479	0.000**
Mindful Eating	1.013	0.153	0.429	6.631	0.000**
Sustainable and Healthy Eating Behaviors	−0.778	0.330	−0.237	−2.360	0.019*

**p < 0.01, *p < 0.05.

In contrast, adult nutrition knowledge had an adverse, statistically significant effect on sustainable and healthy eating behavior (*B* = −0.778; *β* = −0.237; *t* = −2.360; *p* = 0.019). According to the standardized coefficients, diet self-efficacy contributed most to explaining sustainable and healthy eating behavior, followed by mindful eating.

**Figure 5:**
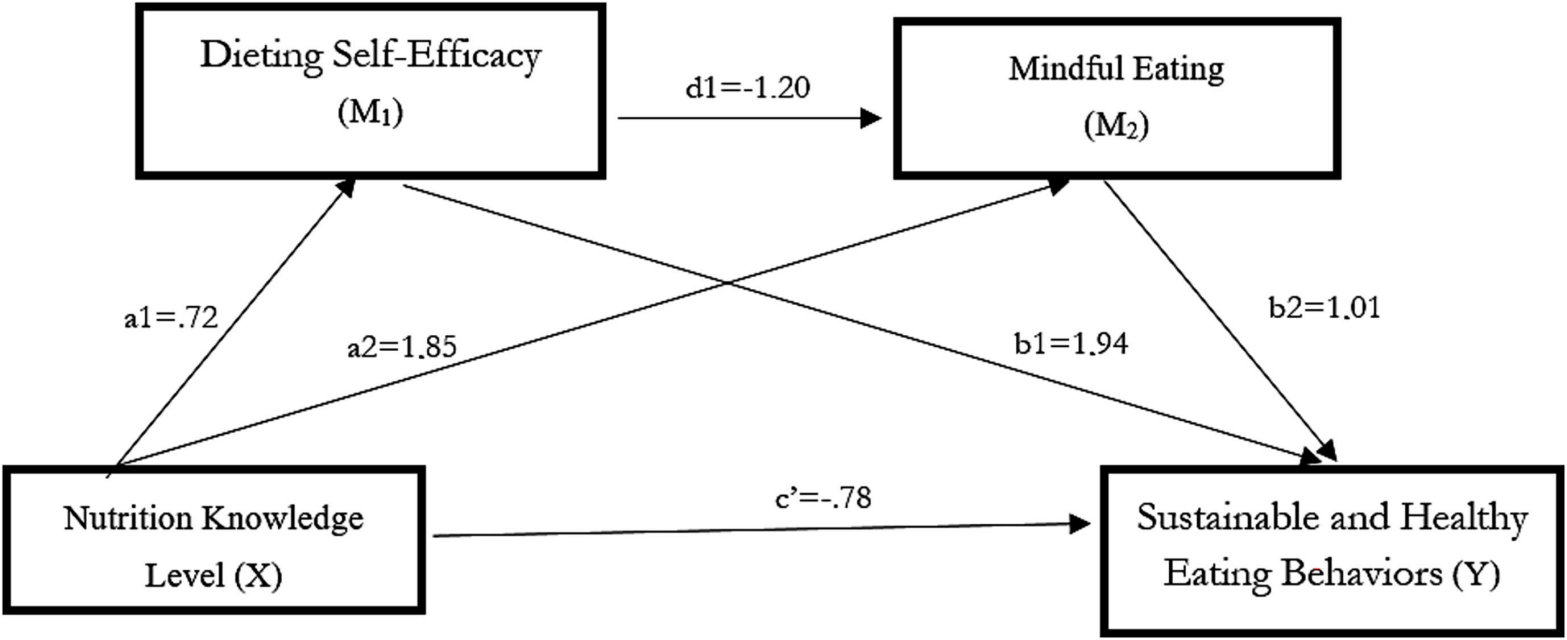
The serial mediation effects of dieting self-efficacy and mindful eating.

As shown in Table 11, nutrition knowledge had a positive and statistically significant effect on diet self-efficacy (path a1) (a1 = 0.72, *t* = 32.18, *p* < 0.001). Diet self-efficacy, in turn, exerted a positive and significant effect on sustainable and healthy eating behaviors (path b1) (b1 = 1.94, *t* = 7.48, *p* < 0.001). Nutrition knowledge also had a positive and statistically significant effect on mindful eating (path a2) (a2 = 1.85, *t* = 43.11, *p* < 0.001). Mindful eating had a positive and significant effect on sustainable and healthy eating behaviors (path b2) (b2 = 1.01, *t* = 6.63, *p* < 0.001). In addition, diet self-efficacy showed a negative and statistically significant effect on mindful eating (path d1) (d1 = −1.20, *t* = −25.70, *p* < 0.001). When the direct association between nutrition knowledge and sustainable and healthy eating behaviors was examined (path c′), it was found to be negative and statistically significant (c′ = −0.78, *t* = −2.36, *p* = 0.02). Figure 5 illustrates the serial mediating effects of diet self-efficacy and mindful eating.

**Table 11:** The serial mediation role of diet self-efficacy and mindful eating on the relationship between nutrition knowledge and sustainable and healthy eating behaviors (*N* = 671).

Outcomes															
	Diet self-efficacy (M_1_)					Mindful eating (M_2_)					Sustainable and healthy eating behaviors (Y)				
		*b*	SE	*t*	*p*		*b*	SE	*t*	*p*		*b*	SE	*t*	*p*
Nutrition Knowledge (X)	a1	0.72	0.02	32.18	0.00	a2	1.85	0.04	43.11	0.00	c’	−0.78	0.33	−2.36	0.02
Diet Self-Efficacy (M_1_)	-	-	-	-		d1	−1.20	0.05	−25.70	0.00	b1	1.94	0.26	7.48	0.00
Mindful Eating (M_2_)	-	-	-	-		-	-	-	-		b2	1.01	0.15	6.63	0.00
Constant		−9.50	1.41	32.75			17.03	1.80	9.45			19.44	7.58	2.57	
		R^2^ = 0.61					R^2^ = 0.75					R^2^ = 0.30			
		*F*(1,669) = 1035.36					*F*(1,669) = 1008.57					*F*(1,669) = 95.81			
		*p* = 0.00**					*p* = 0.00**					*p* = 0.00**			

**p < 0.01.

As illustrated in Figures 5, a serial multiple mediation Model 6 was employed. The model includes two mediating variables, three indirect effects, and one direct effect. These effects are as follows: the indirect association between nutrition knowledge and sustainable and healthy eating behaviors through diet self-efficacy (a^1^b^1^); the indirect association between nutrition knowledge and sustainable and healthy eating behaviors through mindful eating (a^2^b^2^); and the indirect association between nutrition knowledge and sustainable and healthy eating behaviors through diet self-efficacy and mindful eating sequentially (a^1^d^1^b^2^). The sum of these three indirect effects represents the total indirect effect on sustainable and healthy eating behaviors (serial mediation effect: a^1^b^1^ + a^2^b^2^ + a^1^d^1^b^2^).

As shown in Table 12, nutrition knowledge had a positive, statistically significant total indirect effect on sustainable and healthy eating behaviors (*B* = 2.40, SE = 0.36, CI = [1.65, 3.08]). The first indirect effect represents the pathway from nutrition knowledge to sustainable and healthy eating behaviors via diet self-efficacy [Ind1: nutrition knowledge → diet self-efficacy → sustainable and healthy eating behaviors]. This indirect effect was statistically significant (*B* = 1.39, SE = 0.22, CI = [0.94, 1.81]). The second indirect effect reflects the pathway from nutrition knowledge to sustainable and healthy eating behaviors via mindful eating [Ind2: nutrition knowledge → mindful eating → sustainable and healthy eating behaviors]. This indirect effect was also statistically significant (*B* = 1.88, SE = 0.33, CI = [1.22, 2.50]). The third indirect effect represents the serial pathway from nutrition knowledge to sustainable and healthy eating behaviors through diet self-efficacy and mindful eating [Ind3 = nutrition knowledge → diet self-efficacy → mindful eating → sustainable and healthy eating behaviors]. This indirect effect was statistically significant (*B* = −0.87, SE = 0.15, CI = [−1.18, −0.57]).

**Table 12:** The indirect effects of nutrition knowledge on sustainable and healthy eating behaviors.

Indirect effects	*b*	SE	LLCI	ULCI
Total effect	2.40	0.36	1.65	3.08
Ind 1	1.39	0.22	0.94	1.81
Ind 2	1.88	0.33	1.22	2.50
Ind 3	−0.87	0.15	−1.18	−0.57

An important pattern observed in the mediation analysis is that the total association between nutrition knowledge and sustainable and healthy eating behaviors was positive. In contrast, the direct effect became negative when mediators were included in the model. This pattern is indicative of a potential suppression effect, in which the inclusion of mediating variables enhances the model’s predictive validity by accounting for shared variance among predictors (73).

Given the high intercorrelations among the predictor variables, the observed pattern may also reflect statistical suppression, where the inclusion of mediators alters the magnitude and direction of direct effects (73, 74). Suppression effects are particularly likely in models involving conceptually related psychological constructs, as shared variance may obscure or reverse direct associations. Therefore, the regression coefficients and mediation pathways should be interpreted with caution, and future research using latent variable modeling approaches is recommended to disentangle these complex relationships.

In this context, nutrition knowledge appears to influence eating behavior through diet self-efficacy and mindful eating indirectly. At the same time, the residual direct effect after controlling for these mediators becomes negative. This suggests that the relationship between knowledge and behavior is not linear but operates through complex and potentially competing mechanisms.

In addition to the primary analyses, gender was included as a covariate to examine whether the observed relationships remained robust after controlling for biological sex differences. As presented in Table 13, nutrition knowledge did not significantly predict diet self-efficacy (*b* = 0.20, SE = 0.65, 95% CI [−1.07, 1.48]) or mindful eating (*b* = −0.71, SE = 1.02, 95% CI [−2.70, 1.29]) when gender was controlled.

**Table 13:** Regression coefficients for the associations between nutrition knowledge and study variables controlling for gender.

Independent	Depend	*b*	SE	LLCI	ULCI
Nutrition knowledge	Diet self-efficacy	0.20	0.65	−1.07	1.48
Nutrition knowledge	Mindful eating	−0.71	1.02	−2.70	1.29
Diet self-efficacy					
Nutrition knowledge	Sustainable and healthy eating behaviors	−1.10	0.25	−1.60	−0.61
Diet self-efficacy					
Mindful eating					
Nutrition knowledge	Sustainable and healthy eating behaviors	−1.24	0.69	−2.59	0.12

However, nutrition knowledge demonstrated a statistically significant negative association with sustainable and healthy eating behaviors (*b* = −1.10, SE = 0.25, 95% CI [−1.60, −0.61]). In contrast, when examined within the full model, the direct association between nutrition knowledge and sustainable and healthy eating behaviors was no longer statistically significant (*b* = −1.24, SE = 0.69, 95% CI [−2.59, 0.12]).

These findings suggest that including gender as a covariate does not substantially alter the overall pattern of results but may contribute to variability in the strength and significance of specific pathways.

## 4 Discussion

While the proposed theoretical framework assumed a sequential and predominantly positive pathway among variables, the empirical findings partially diverged from these expectations. This discrepancy highlights the importance of data-driven interpretation. It suggests that theoretical assumptions regarding linear mediation processes in eating behavior may require refinement. Such inconsistencies are not uncommon in behavioral research and often indicate the presence of more complex, context-dependent mechanisms (25).

From a nutrition science perspective, sustainable and healthy eating behaviors should be interpreted within established dietary frameworks, including dietary guidelines and food processing classifications. For example, the NOVA classification system emphasizes the health and environmental implications of consuming ultra-processed foods (75). Similarly, global dietary recommendations highlight plant-based dietary patterns as key components of sustainable diets (1). Integrating such frameworks into behavioral models enhances the applicability of findings to public health nutrition and policy contexts.

This study examined the serial mediating roles of diet self-efficacy and mindful eating in the relationship between nutrition knowledge and sustainable and healthy eating behaviors among sports science students. The findings indicate that nutrition knowledge exerts both direct and indirect effects on sustainable and healthy eating behaviors; however, this relationship is mainly shaped by psychosocial mechanisms.

This study contributes to sustainability literature by proposing a micro-level behavioral pathway through which cognitive resources (nutrition knowledge) are transformed into sustainability-relevant consumption behaviors via self-regulatory psychological mechanisms. By integrating Social Cognitive Theory and mindfulness-based approaches, the model advances sustainability behavior research beyond attitudinal explanations and highlights the importance of internal capacities in enabling responsible consumption.

The initial results of the study revealed a moderate, positive association between nutrition knowledge and sustainable, healthy eating behaviors. Similarly, Öngün Yılmaz et al. (40) reported that nutrition knowledge is a significant determinant of sustainable and healthy eating behaviors and that as knowledge levels increase, individuals are more likely to engage in environmentally and health-oriented food choices. Previous research has likewise demonstrated that higher levels of knowledge and literacy regarding food composition, diet–health relationships, and principles of sustainable nutrition are associated with a greater likelihood of exhibiting conscious eating behaviors that take environmental impacts into account (76, 77).

Nevertheless, the regression analyses indicated that nutrition knowledge alone explained sustainable and healthy eating behaviors only to a limited extent. This finding is consistent with the widely discussed knowledge behavior gap in the literature. Indeed, previous studies have shown that nutrition-related knowledge and attitudes are not consistently translated into behavior and that additional psychosocial mechanisms are required to facilitate behavior change (7, 8).

The reversal of the direct association with nutrition knowledge when mediators are introduced suggests that the relationship between knowledge and behavior is conditional upon underlying psychological processes. This finding aligns with previous research indicating that knowledge alone may not directly translate into behavior unless supported by motivational and self-regulatory mechanisms (7).

One possible explanation is that individuals with higher levels of nutrition knowledge may experience increased cognitive awareness of dietary ideals, which, in the absence of sufficient self-regulatory capacity, may lead to inconsistencies or even disengagement from behavior (78). Thus, the negative direct effect should not be interpreted as detrimental, but rather as reflecting the necessity of mediating mechanisms in enabling knowledge-driven behavior change.

The serial mediation analyses further revealed that nutrition knowledge had a strong and positive effect on diet self-efficacy. When interpreted within the framework of Social Cognitive Theory, this result suggests that knowledge levels strengthen individuals’ self-efficacy beliefs regarding their ability to initiate and maintain healthy eating behaviors. Individuals with higher levels of nutrition knowledge tend to feel more competent when making food choices, which, in turn, increases the likelihood that this knowledge will be translated into action.

The finding that diet self-efficacy is significantly and positively associated with sustainable and healthy eating behaviors is consistent with theoretical and empirical evidence supporting the central role of self-efficacy in behavior change and maintenance. According to Social Cognitive Theory, self-efficacy is a fundamental psychosocial determinant reflecting individuals’ beliefs in their capacity to initiate, sustain, and persist in a given behavior despite obstacles (12). Research in the nutrition domain has demonstrated that self-efficacy for healthy eating is strongly and consistently associated with fruit and vegetable intake, diet quality, and the maintenance of healthy dietary behaviors (14). Moreover, in contemporary models aimed at explaining sustainable eating behaviors, self-efficacy is conceptualized as a critical mechanism facilitating individuals’ translation of environmental and health-oriented dietary goals into everyday practices (79).

When considering the context of sports science students, who undergo education emphasizing both performance and health beliefs in their ability to sustain healthy eating behaviors even under intense academic and physical demands, emerges as a decisive factor supporting behavioral continuity.

One noteworthy finding of the study is a negative association between diet self-efficacy and mindful eating. This finding suggests that individuals with high self-efficacy may regulate their eating behaviors through more automated, goal- and outcome-oriented strategies. As emphasized in Bandura’s (12) Social Cognitive Theory, a strong sense of self-efficacy can facilitate the maintenance of behaviors with reduced cognitive effort, allowing actions to become more habit-based. In this context, individuals’ strong beliefs in their ability to “successfully manage” their behavior may shift their focus from the process itself to the desired outcome.

An alternative explanation for this finding may lie in the distinction between controlled and automatic forms of self-regulation. High levels of diet self-efficacy may promote structured, goal-oriented eating strategies that rely on planning and cognitive control rather than present-moment awareness (57). In contrast, mindful eating emphasizes non-judgmental awareness and experiential engagement with food (19).

Thus, these constructs may represent partially divergent self-regulatory pathways, where increased reliance on control-based strategies may attenuate mindfulness-related processes. This interpretation highlights the multidimensional nature of eating regulation and suggests that different psychological mechanisms may not always operate synergistically.

The negative association observed between diet self-efficacy and mindful eating, as well as the negative direct association between nutrition knowledge and sustainable and healthy eating behaviors, warrants careful theoretical reconsideration. Although the dominant literature suggests positive associations among these constructs (12, 14), emerging evidence indicates that higher levels of perceived control and self-regulation may, under certain conditions, lead to more rigid and goal-oriented eating patterns that are less compatible with mindfulness-based awareness (20, 56).

Specifically, individuals with elevated diet self-efficacy may rely more on automatic, rule-based dietary strategies (e.g., calorie monitoring, performance-driven eating), thereby reducing attentional engagement with the sensory and experiential aspects of eating. This interpretation aligns with dual-process models, suggesting that highly controlled behaviors may operate with reduced conscious awareness over time (57).

Furthermore, the negative direct association between nutrition knowledge may reflect a suppression effect, in which the inclusion of mediators reverses the direction of the association (73). In such cases, knowledge alone may not translate into behavior and may even be associated with cognitive overload, ambivalence, or unrealistic dietary expectations (7). Therefore, these findings should not be interpreted as contradictory per se, but rather as indicative of complex and potentially competing psychological processes underlying eating behavior.

Importantly, the present findings reveal a notable divergence between the theoretically proposed positive and sequential pathway and the empirically observed pattern. Specifically, the negative association between diet self-efficacy and mindful eating, along with the negative serial indirect effect, suggests that the hypothesized linear mechanism may not fully capture the complexity of the underlying psychological processes. Rather than representing a straightforward facilitative pathway, the results indicate the presence of competing or partially opposing self-regulatory mechanisms operating simultaneously.

This pattern is consistent with emerging perspectives in health psychology suggesting that different forms of self-regulation, such as control-based (self-efficacy-driven) versus awareness-based (mindfulness-driven) processes, may not always function synergistically (57, 80). Instead, individuals with high perceived control may rely more on structured, goal-oriented strategies, which could attenuate experiential awareness during eating.

Therefore, the current findings necessitate a reinterpretation of the proposed model, emphasizing that the relationship between nutrition knowledge and eating behavior is not strictly linear but operates through complex, and at times competing, psychological pathways.

In contrast, mindful eating is a process that requires awareness of the eating moment, present-moment attention, and sensitivity to bodily hunger satiety cues rather than an emphasis on behavioral success (18, 19). While individuals with high self-efficacy may manage their eating behaviors in a more controlled and planned manner, they may pay less attention to the sensory and internal experiences involved in the eating process. Indeed, the literature suggests that highly controlled, performance-oriented eating approaches may attenuate the mindfulness component of eating behavior (20).

The positive association between mindful eating and sustainable, healthy eating behaviors underscores the critical role of eating awareness for both individual health and sustainability. By encouraging individuals to focus not only on what they eat but also on why and how they eat, mindful eating reduces automaticity in eating behavior and thereby limits tendencies toward overeating and emotional eating (18, 19).

Mindfulness-based eating approaches promote reliance on internal hunger satiety cues and experiential awareness rather than food choices driven by environmental stimuli such as advertising, portion sizes, or social contexts (20). In this respect, mindful eating not only supports healthy dietary practices but also strengthens sustainable eating behaviors by reducing excessive consumption, fostering more balanced food choices, and encouraging the adoption of plant-based dietary patterns (24, 54).

Eating behavior is shaped by multi-level determinants, including environmental, economic, and cultural factors (81). The absence of such contextual variables in the present model limits the ecological validity of the findings.

The findings from the serial mediation model indicate that the association between nutrition knowledge and sustainable and healthy eating behaviors is primarily mediated by diet self-efficacy and mindful eating. The significance of the total indirect effect and the reversal in the direction of the direct effect when mediators are included in the model clearly demonstrate the decisive role of psychosocial processes in translating nutrition knowledge into behavior. These results suggest that nutrition education should not be limited to information provision alone but should also be structured to foster self-efficacy development and support mindful eating skills.

The additional analyses controlling for gender provide further insight into the robustness of the proposed model. Although gender did not substantially alter the overall pattern of relationships, the attenuation of certain effects suggests that biological sex may moderate or confound the association between nutrition knowledge and eating behaviors.

Previous research has consistently demonstrated gender differences in dietary patterns, eating regulation, and health-related behaviors, with women generally reporting higher levels of dietary restraint, health consciousness, and nutritional awareness compared to men (82, 83). These differences may influence how nutrition knowledge is internalized and translated into behavior.

The non-significant associations between nutrition knowledge and both diet self-efficacy and mindful eating, even after controlling for gender, may indicate that these relationships are partially dependent on gender-specific psychological or behavioral tendencies. Therefore, future studies should better test gender as a moderator within structural models to capture potential differential pathways (84).

The relatively high intercorrelations among the study variables raise the possibility of multicollinearity and suppression effects, which may influence the stability and interpretability of regression coefficients (72). In particular, suppression may explain the observed reversal in the direction of the direct association between nutrition knowledge. Future studies employing structural equation modeling (SEM) may provide more robust estimates by accounting for measurement error and latent structures.

## 5 Conclusion

The findings of this study indicate that nutrition knowledge is associated with sustainable, healthy eating behaviors, both directly and indirectly through psychological mechanisms. However, given the cross-sectional design, these relationships should be interpreted as associative rather than causal. The findings indicate that nutrition knowledge provides a necessary cognitive foundation for sustainable dietary practices; however, translating this knowledge into behavior predominantly occurs through individuals’ self-efficacy beliefs regarding their ability to maintain healthy eating and through their levels of awareness during the eating process.

The serial mediation analyses revealed that the direct association between nutrition knowledge weakened and changed direction when the mediating variables were included in the model, thereby clearly underscoring the decisive role of psychosocial mechanisms in the knowledge–behavior relationship. These results suggest that sustainable and healthy eating is shaped not only by what individuals know but also by the extent to which that knowledge is rendered actionable.

In conclusion, interventions designed to promote sustainable eating behaviors should move beyond knowledge dissemination alone and instead incorporate holistic approaches that strengthen diet self-efficacy and cultivate mindful eating skills.

Furthermore, including gender as a covariate suggests that, while the overall model remains stable, gender-related differences may influence specific pathways linking nutrition knowledge to eating behaviors. This highlights the importance of considering demographic variables in the design of future interventions.

From a sustainability standpoint, these findings indicate that students with higher nutrition knowledge are more likely to engage in environmentally and health-conscious eating behaviors when supported by psychological self-regulation mechanisms. This suggests that sustainable food consumption is not merely a function of environmental awareness but emerges through a sequence of cognitive and behavioral competencies.

Overall, the findings of the present study contribute to the literature by demonstrating that the relationship between nutrition knowledge and sustainable eating behaviors is not direct but operates through sequential psychological mechanisms. This supports contemporary behavioral models suggesting that knowledge-based interventions are insufficient unless complemented by strategies targeting self-efficacy and self-regulation (16, 50).

The identification of both positive indirect effects and a negative direct effect further underscores the complexity of behavioral processes. It highlights the importance of adopting integrative, multi-pathway models in nutrition and sustainability research.

## 6 Limitations of the study

The findings of this study should be interpreted in light of several limitations. First, the research employed a cross-sectional design, and data were collected at a single point in time. Therefore, the relationships among the variables cannot be interpreted causally. Although the proposed serial mediation model linking nutrition knowledge, diet self-efficacy, and mindful eating is grounded in strong theoretical foundations, the observed mediation effects represent statistical mediation rather than causal processes. Future studies are encouraged to employ longitudinal or experimental designs to test the temporal and causal relationships among these variables.

Although the cross-sectional design of this study precludes causal inference, it is well suited for theory-building in sustainability research, where identifying psychologically plausible behavior pathways constitutes a critical first step. The proposed serial mediation model provides an empirically grounded framework that can inform future longitudinal and intervention-based sustainability studies aimed at promoting sustainable food consumption. Given the cross-sectional design, causal interpretations are not warranted; the findings should be interpreted as associative rather than causal (27).

Sustainable eating in the present study was operationalized primarily through health-oriented and responsibility-based dietary behaviors. While this approach captures an important dimension of sustainability, future research should incorporate objective environmental indicators, such as carbon footprint, food sourcing practices, and waste-reduction behaviors.

Second, the sample was limited to sports science students and was recruited using convenience sampling. Although Monte Carlo simulation results indicated that the sample size provided sufficient statistical power for the proposed serial mediation model, the relatively high levels of health awareness and physical activity typical of sports science students may limit the generalizability of the findings to students from other faculties or to the general adult population.

Third, an important limitation is reliance on self-reported measures to assess sustainable and healthy eating behaviors. Self-report instruments are subject to biases such as social desirability and recall bias, which may lead to overestimation of healthy behaviors (85). Moreover, the absence of objective dietary assessment methods (e.g., 24-h dietary recalls, food frequency questionnaires, or diet quality indices such as the Healthy Eating Index) limits the ability to determine actual dietary intake (86). Therefore, the findings of the present study should be interpreted as reflecting perceived rather than objectively measured eating behaviors.

Fourth, sustainable and healthy eating behaviors were assessed through subjective self-report measures, and objective indicators, such as actual food intake, portion sizes, and environmental metrics (e.g., carbon and water footprints), were not included in the study. Consequently, the extent to which the reported behaviors correspond to actual environmental impacts cannot be determined with certainty. Although gender was included as a covariate in additional analyses, the study did not explicitly test gender-based moderation effects. Future research employing multigroup or moderation analyses is warranted to understand gender-specific mechanisms better.

Finally, although sustainability is a multidimensional construct, the analyses focused primarily on individual dietary behaviors, and contextual factors such as the food environment, economic conditions, cultural norms, and structural determinants were not incorporated into the model. Moreover, not all components of the Theory of Planned Behavior (i.e., attitudes, subjective norms, and perceived behavioral control) were comprehensively assessed. Future research employing multilevel models that integrate individual psychological processes with environmental and contextual variables would contribute to a more holistic understanding of sustainable and healthy eating behaviors.

## 7 Practical implications

The findings of this study offer important implications for practices aimed at promoting sustainable and healthy eating behaviors. First, the results indicate that nutrition knowledge alone is insufficient to explain sustainable and healthy eating behaviors, and that psychosocial mechanisms such as diet self-efficacy and mindful eating play a decisive role in translating knowledge into action. Therefore, nutrition education programs for university students are recommended to move beyond mere information provision and to incorporate interventions that strengthen individuals’ self-efficacy beliefs regarding their ability to maintain healthy dietary practices.

From a sustainability perspective, the findings suggest that interventions to promote sustainable food consumption among university students should move beyond information-based nutrition education. Programs integrating self-efficacy enhancing strategies (e.g., skill-based meal planning, affordable, sustainable food choices) and mindfulness-based eating practices may more effectively support responsible consumption patterns. Universities and sport science faculties can incorporate mindful eating modules into health and performance curricula, thereby fostering dietary behaviors that simultaneously support personal well-being and environmental sustainability.

Moreover, given the positive effects of mindful eating on sustainable and healthy eating behaviors, integrating mindfulness-based eating awareness training into university curricula or campus-based health programs may be beneficial. Such interventions could help students become more sensitive to internal hunger satiety cues, reduce excessive and impulsive consumption, and make food choices that take environmental impacts into account.

Particularly for sports science students who are likely to assume guidance roles in health and performance domains in the future, fostering sustainable and healthy eating behaviors is of strategic importance. Accordingly, programs targeting this group should emphasize not only individual health benefits but also environmental responsibility and the role-modeling dimension of dietary practices.

## 8 Suggestions for future research

Future studies may benefit from incorporating socioeconomic and dietary pattern variables as potential moderators of sustainable eating behaviors.

Future research should re-examine the serial mediation relationships tested in this study using longitudinal and experimental designs in order to clarify the temporal and causal links among the variables. In particular, investigating how the interactions among nutrition knowledge, diet self-efficacy, and mindful eating evolve is of considerable importance.

Moreover, because this study was limited to sports science students, future research conducted among students from different faculties, the general adult population, or across diverse cultural contexts is recommended to test the generalizability of the proposed model. Incorporating contextual factors such as cultural norms, economic conditions, and the food environment into the model may enable a more comprehensive understanding of sustainable and healthy eating behaviors.

In addition, future studies are encouraged to assess sustainable and healthy eating behaviors not only through self-report measures but also by incorporating objective indicators, such as actual food intake data, diet quality indices, and environmental metrics, including carbon and water footprints. This approach would provide clearer insight into the extent to which reported behaviors correspond to real environmental impacts.

Finally, testing multivariate and multilevel models that encompass all components of the Theory of Planned Behavior (i.e., attitudes, subjective norms, and perceived behavioral control) alongside diet self-efficacy and mindful eating would contribute to a more detailed explanation of the psychological processes underlying sustainable and healthy eating behaviors.

## 9 Policy-level implications

At the policy level, this study’s findings highlight the need for integrated, psychologically informed strategies to promote sustainable food consumption in higher education institutions. The results indicate that nutrition knowledge alone is insufficient to foster sustainable eating behaviors unless it is supported by internal capacities such as dietary self-efficacy and mindful eating. This suggests that sustainability-oriented food policies should move beyond informational campaigns and incorporate skill-building and self-regulation components.

Universities, particularly faculties of sport sciences, can play a pivotal role in advancing sustainable consumption by embedding self-efficacy enhancing nutrition education and mindful eating practices into institutional curricula. Such policies may include experiential learning approaches, campus-based sustainable food initiatives, and structured programs that empower students to make responsible dietary choices aligned with both personal health and environmental sustainability.

From a broader sustainability governance perspective, these findings support the development of multi-level policies that align educational institutions with national and global sustainability agendas, including the United Nations Sustainable Development Goals related to responsible consumption and well-being. By fostering psychological mechanisms that facilitate sustainable dietary behavior, universities can act as key agents in sustainability transitions, contributing to long-term societal change by cultivating environmentally responsible future professionals. Such policy initiatives may be operationalized through curriculum-integrated sustainability modules, campus food-environment regulations, and interdisciplinary collaborations among health, nutrition, and sustainability units.

## Data Availability

The original contributions presented in the study are included in the article/supplementary material, further inquiries can be directed to the corresponding authors.
